# Book Reviews

**Published:** 1905-06

**Authors:** 


The Influence of Growth on Congenital and Acquired Deformities. By Adoniram Brown Judson, M.D., Orthopedic Surgeon to the Out-Patient Department, New York Hospital, 1878-1903. Octavo, pp. 286. Illustrated. New York: William Wood & Company. 1905.

This author presents in this book not a new subject nor yet a novel invention, but discusses in a refreshing manner the effect of growth on deformity. Stated in another way, Judson, in this volume, accentuates the fact that the prevention and cure of deformities rest largely in the management of cases that natural growth will work for their recovery. Indeed, it is believed by the author that this is, or should be made, the principal factor of treatment.

To cure a child that is deformed, and thus convert a dependent being into a selfsupporting individual, is one of the highest functions of a physician. It is this that entitles the orthopedic surgeon to the gratitude, not only of his patients, but of all mankind. In this monograph is found the most modern thought by an experienced orthopedist, on a phase of the work not usually discussed in works on deformities.

Mr. Hilton's aphorism on the necessity of rest to accomplish growth and repair is accentuated by Judson and made to play an important rôle in his effort to cure maimed and distorted infants and youth. That portion of this treatise devoted to the consideration of hip disease could be read with profit by every physician and so, for that matter, could the chapters on Pott's disease and on lateral curvature of the spine.

It is, by and large, one of the most important monographs on deformities issued in many years and is, withal, beautifully printed and superbly illustrated.

Practical Pediatrics. A Manual of the Medical and Surgical Diseases of Infancy and Childhood. By Dr. E. Graetzer, Editor of the *Centralblatt fur Kinderheilkunde*. Authorised translation. By Herman B. Sheffield, M.D., Instructor in Diseases of Children, and Attending Pediatrist, New York Post-Graduate Medical School and Hospital. Octavo. Pages xii.-544. Philadelphia: F. A. Davis Company. 1905. (Cloth, $3.00.)

It is quite surprising, now that attention is directed to the fact to learn that a reference book on pediatrics has not existed until this one was put forth. The author and translator have performed a valuable service to the profession in arranging in compact form a working manual of such excellent quality.

The book is rightly named for it is, indeed, practical in character. In the nineteen chapters that comprise part one, are told the essentials of pediatric science from the care of the newborn to the management of the most intricate disorders of nutrition, or of the special senses. The author's directions relating to physical diagnosis are excellent and his management of the diseases of the newly born commends itself to the better judgment of the physician of experience.

It is a book, moreover, that will find favor with every young physician who is called upon to treat infants or young children. It will guide him through many of the difficulties he is sure to meet and will prove a safe counselor whenever he is in doubt.

---

Transactions of the Twenty-sixth Annual Meeting of the American Laryngological Association, held at Atlantic City, N. J., June 2, 3 and 4, 1904. James E. Newcomb, M.D., Secretary. New York: Rooney & Otten Printing Co.

The association which creates this volume of transactions each year is noted for the excellence of its work, and its published volumes constitute an expression of the best thought on topics pertaining to diseases of the nose and throat. The issue for 1904 now before us, contains thirty-one scientific papers, not including the president's address or the supplementary material such as the official minutes and reports of officers. Every subject dealt with is of absorbing interest to the laryngologist, and some of them are of interest and value to the general practitioner. Very few society transactions of 350 pages present a more acceptable showing than does this one, and it is a book that will be consulted as authoritative on the topics discussed.

---

The International Medical Annual. A Yearbook of Treatment and Practitioners' Index. Thirty-five contributors, American and Foreign. Twenty-third year, 1905. Octavo, pp. 675. New York: E. B. Treat & Company. (Price, $3.00.)

The annual for this year appears in larger size than usual. This is rendered necessary by the increase in the material. It now assumes a better proportion, the larger page being in keeping with the increase in bulk.

It contains an enormous mass of well digested and splendidly arranged literature, and is one of the most serviceable of the yearly reference books. We think the editor should no longer withhold his name from such a creditable work. The names of the contributors are given: why should not that of the editor likewise be presented?

We commend this edition as the very best of this most excellent series. It grows better year by year.

